# Trail marking by larvae of the cactus moth, *Cactoblastis cactorum*

**DOI:** 10.1093/jis/14.1.64

**Published:** 2014-01-01

**Authors:** Terrence D. Fitzgerald, Michael Wolfin, Frank Rossi, James E. Carpenter, Alfonso Pescador-Rubio

**Affiliations:** 1 Department of Biological Sciences, State University of New York at Cortland, Cortland, NY 13045, USA; 2 Department of Chemistry, State University of New York at Cortland, Cortland, NY 13045, USA; 3 James Carpenter, U.S. Department of Agriculture Research Service, Crop Protection and Research Laboratory, Tifton, Georgia, USA; 4 Centro Universitario de Investigación y Desarrollo Agropecuario, Universidad de Colima, Tecomán 28100, Colima, México

**Keywords:** mandibular glands, social caterpillar, silk, trail pheromone, cactus caterpillar, 2-acyl-1, 3 cyclohexane dione

## Abstract

The cactus moth, *Cactoblastis cactorum* (Berg) (Lepidoptera: Pyralidae), spends most of its larval life feeding within the cladodes of *Opuntia* cactuses, but the gregarious caterpillars begin their life outside the plant, and in the later instars make intermittent excursions over plant surfac- es to access new cladodes and to thermoregulate. The study reported here showed that when the caterpillars move *en masse* , they mark and follow trails that serve to keep the cohort together. Artificial trails prepared from hexane extracts of the caterpillar’s paired mandibular glands were readily followed by the caterpillars. The glands are remarkably large, and their fluid contents, which constitute approximately 1% of the total wet mass of a caterpillar, are secreted onto the substrate as they move. Although the caterpillars also lay down copious quantities of silk, the ma- terial in itself neithxer elicits trail following nor is it a requisite component of pathways that elicit trail following. Previous analyses of the mandibular glands of other species of pyralid caterpillars showed that they contain a series of structurally distinct 2-acyl-1,3 cyclohexane diones. Chemical analysis indicates that the glands of *C. cactorum* contain structurally similar compounds, and bio- assays indicate that trail following occurs in response to these chemicals. While the mandibular glands’ fluids have been shown to act as semiochemicals, effecting both interspecific and intra- specific behavior in other species of pyralids, the present study is the first to report their use as a trail pheromone.

## Introduction

The larvae of *Cactoblastis cactorum* (Berg) (Lepidoptera: Pyralidae) are internal feeders on cactuses, most notably those occurring in the prickly pear genus *Opuntia* . The insect is best known for bringing under control the runaway growth of prickly pear in Australia ( Raghu and Walton 2007 ). In 1989, the insect was discovered in Florida feeding on native species of *Opuntia* ( Habeck and Bennett 1990 ; Dickel 1991 ; Stiling and Moon 2001 ). Since then, the range of the caterpillar has expanded westward to Louisiana ( Rose et al. 2011 ), and there is concern that it may eventually reenter Mexico, where a small population on Isla Mujeres, Quintana Roo, was eradicated in 2007 ( NAPPO 2008 ). In Mexico, *Opuntia* is not only an ecologically significant member of the native flora but also an agriculturally important species.

Although *C. cactorum* has been the subject of numerous studies due to its success as a control agent for invasive species of *Opuntia* cactuses, and its more recent status as an invasive species itself, little is known of the fine structure of the foraging behavior of the caterpillars. Indeed, the larvae are found mostly within cladodes, where their behavior is largely unobservable. Pettey (1947) and Zimmerman et al. (2004), however, confirmed Dodd’s (1940) observation that, following the depletion of the contents of a cladode, colonies may move to a new cladode by traveling over the surface of the host plant and that colonies divided and wandered over the plant surface when they were from one-half to two- thirds grown. Colonies Monro (1967) observed required on average four cladodes to complete their development. The newly eclosed caterpillars may also fail to enter a cladode at the oviposition site, due to either cuticular toughness or sticky exudates, forcing the small caterpillar to move *en masse* over the surface of the plant to a new site ( Pettey 1947 ; Zimmerman et al. 2004). In addition, the caterpillars move from the interior of a cladode to the outside to seek shade on hot days and may bask outside the cladode on cold days ( Dodd 1940 ; Pettey 1947 ; Zimmerman et al. 2004).

The possibility that *C. cactorum* caterpillars mark and follow chemical trails during the time they spend on the surface of the plant was brought to the attention of the senior author by an illustration of a cohort of the caterpillars moving together over a plant, behavior suggestive of trail following, that accompanied a paper by Raghu and Walton (2007) . To our knowledge, Dodd (1940) is the only investigator to have previously recorded trailing behavior. He noted that the caterpillars may travel over the surface of a plant “…roughly in single file, and the course of the wandering is indicated by a thin trail of silk which furnishes a foothold on the smooth surface of the prickly-pear.”

These and other previous published observations of the biology of *C. cactorum* indicate that the caterpillars are remarkably social, spending the entirety of their larval lives in close proximity. The potential role of semiochemicals in the expression and maintenance of this sociality has not been previously investigated. As a first step, we report here a study of trail following behavior by the caterpillars. Preliminary results confirmed that the caterpillars marked and followed trails, which led to additional investigations to determine the source of the marker, its identity, and the means by which it is deployed by the caterpillars.

## Materials and Methods

### Source of caterpillars and rearing procedure

*C. cactoblastis* egg masses were obtained from the U.S. Department of Agriculture Research Service, Crop Protection and Research Laboratory, Tifton. Georgia, USA. The caterpillars were maintained on an artificial diet according to the procedures of Marti et al. (2008) .

### Trail following behavior

Studies were conducted to determine if *C. cactorum* caterpillars deposit a marker that elicits trail following from conspecifics. To obtain pathways over which caterpillars had walked, a strip of chromatography paper (36 cm long x 3 mm wide) was suspended vertically from its top end. Thirty second-instar caterpillars were placed at the bottom of the strip, one after the other, and allowed to move up the strip *ad libitum* . When the caterpillars reached the top of the strip, they were collected and transferred back to the bottom of the strip and allowed to climb again. This procedure was followed for approximately 20 minutes. At the end of this period the caterpillars were removed and the strip was cut into 2.5 cm long sections. A section was then laid out on a horizontal surface to form one arm of a Y-maze. A blank section of chromatography paper of the same dimensions was used to form the other arm. The stem of a maze was made from either a blank section of paper or from the strip the caterpillars had walked up, alternating for each replicate of the test. A se- cond-instar larva was then placed at the base of the stem of the maze, and its choice of arm was recorded. This procedure was replicated with 10 different caterpillars. Paper sections used to construct the mazes were used only once. To avoid a potential positional bias, the side of the maze on which the blank arm was placed was switched for each replicate of the test.

### Persistence of the trail marker

To assess the persistence of the trail marker, the above procedure was followed, with the exception that 20 second-instar caterpillars were used to establish pathways on suspended strips of chromatography paper as described above. Four different strips were prepared, each with different groups of 20 caterpillars. Response to the trail deposited on one of these strips was tested immediately after its deposition. Response to trails deposited on the other strips was tested after they had aged 24, 48 or 120 hours. In each case, the strips were cut into 2.5 cm sections and paired with blank strips to form Y-mazes as described above. While aging, sections were stored in Petri plates at approximately 21 ± 2°C and exposed to the normal 16:8 L:D fluorescent light regime of the laboratory. For each aging period, Y-maze tests were replicated 14 times, each with a different second-instar caterpillar. Sections used to construct the mazes were used only once, and arms were alternated to preclude a positional bias.

### Response of caterpillars to silk

The caterpillars were observed to spin copious quantities of silk as they moved up the suspended paper strips. Similar silk spinning behavior has been reported for field colonies moving outside the plant ( Dodd 1940 ). Two different methods were employed to determine if silk elicited trail following. Using a previously detailed procedure (Pescador et al. 2011), silk was pulled directly from a caterpillar’s spinneret and wound onto a strip of paper card (3 cm long x 3 mm wide). To obtain the silk, a fifth-instar caterpillar was held between thumb and forefinger, and the tip of its spinneret was touched to the strip of card, which was mounted and slowly rotating in the chuck of a motorized stirrer. The caterpillar was moved back and forth along the length of the strip until the rotating paper was thoroughly covered with silk. Caterpillars readily yielded long, unbroken lengths of silk. Using this procedure, silk was collected from 10 different caterpillars. These silk bearing strips were then paired with blank strips to conduct Y- maze tests with second-instar caterpillars as described above.

The importance of silk to trail following was also assessed by cauterizing the spinnerets of seven fifth-instar caterpillars with a hot pin to prevent them from producing silk. The caterpillars were allowed to recover for 24 hours, after which they were observed under a dissecting microscope to be certain that they were unable to spin silk. The caterpillars were allowed to walk up vertically suspended strips of chromatography paper, which were then cut into sections and paired with blank sections to form Y-mazes as detailed above. Caterpillars were observed to determine if they chose the silk-less maze arm previously walked on by caterpillars or the blank arm. The Y-maze test was replicated 12 times each with a different second-instar caterpillar employing the procedure detailed above.

### Response of caterpillars to cuticular residue

Previous studies of trail marking caterpillars show that most species mark pathways with a trail pheromone deposited when they drag the ventral surfaces of their abdomens along the substrate and deposit surface residue (Fitzgerald and Costa 1999). A study was conducted to determine if *C. cactorum* also marks in this manner. A sheet of 9 cm diameter filter paper was folded in half, and the folded edged was used to collect residue by stroking it along the ventral surface of the abdomen of a fifth-instar caterpillar. Abdominal areas between the extreme posterior tip and the fifth abdominal segment were stroked to collect residue. The paper was then unfolded and laid flat, and the response of a second- or third-instar caterpillar to the residue “trail” was observed directly. The procedure was replicated 10 times. Papers used to collect the residue, fifth-instar caterpillars from which the residue was collected, and test caterpillars were used only once. A response was considered positive if the test caterpillars walked along the whole length of the stroked sections of the papers.

### Response of caterpillars to whole body extract

**Figure 1.**
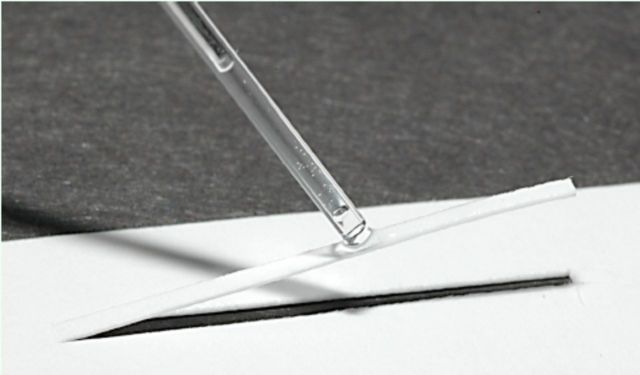
Procedure for establishing chemical-extract and sol- vent-control trails. High quality figures are available online.

An extract was prepared by grinding 10 third- instar caterpillars in hexanes. The extract was placed in a tube and centrifuged. Following centrifugation, the supernatant was decanted and evaporated to dryness. The residue was then dissolved in 1 mL of hexanes. To conduct a bioassay of the extract, the solution was applied in 5 µL quantities to strips of card (2 cm long x approximately 1.5 mm wide) to form artificial trails. The strips were prepared by cutting from a paper card the two long sides of the strip. The other end of the strip. still attached to the card, acted as a hinge as the strip was lifted out of the groove to elevate it above the body of the card. The extract was then applied to the elevated strip to concentrate it on the strip and prevent it from running over into adjacent areas of the card ( Figure 1 ). After allowing five minutes for the solvent to fully evaporate, the strip was set back into the groove so it was flush with the surrounding card. The back side of the strip was taped to the card so that the strip was fixed into position. To conduct a bioassay, a first- or second-instar caterpillar was placed at one end of the marked strip, and a positive response was recorded if it followed the artificial trail to its distant end. As a control, strips marked only with the solvent were also tested. Treatment and control tests were replicated 10 times with different caterpillars. Strips were used only once.

The importance of silk to trail following was also investigated in another study. The response of caterpillars given a choice in Y-maze tests to 2 cm-long arms marked with 5 µL of the extract and arms wrapped with silk pulled directly from the spinneret as described above was assessed. For these Y-maze tests, extract was prepared by soaking 50 second-instar caterpillars in hexane for one hour, evaporating the sample to dryness and reconstituting it to a total volume of 1 mL with hexanes. The test was replicated 12 times with different second- instar caterpillars. Mazes were used only once, and the position of the arms bearing silk and extract were alternated to avoid a potential positional bias.

### Response of caterpillars to crude mandibular gland extract

Following the observation that caterpillars followed trails made from whole body extracts, studies were conducted to localize the source of the pheromone. Systematic bioassays of extracts of external and internal components of the caterpillar eventually led to the mandibular glands. Studies of other pyralid caterpillars in the subfamily Phycitinae have shown that the larvae have prominent mandibular glands that secrete an oily fluid (Mudd and Corbet 1973 ; Mossadeigh 1978, 1980; Mudd 1978 , 1981, 1983; Howard and Blake 2004 ). Though previously unreported, it was determined by dissection that mandibular glands also occur in the larvae of *C. cactorum* , and a study was conducted to determine if an extract of the glands elicited trail following. Each of 10 fifth-instar caterpillars was killed by freezing, weighed to determine its wet body mass, then dissected in physiological saline to remove the paired mandibular glands. To obtain an estimate of the amount of oil contained in the glands, pairs of glands were placed on preweighed sections of filter paper, approximately 2 mm2, and the glands allowed to dry for one hour. The papers were then reweighed. This procedure allowed water clinging to the glands to evaporate and the walls of the glands to dry, largely reducing them to their oily contents. The ratio of each pair of glands to the total wet body mass of the caterpillar from which they were dissected was calculated. The papers bearing the 10 pairs of glands were then placed, collectively, in 1 mL of hexane, vortexed, and allowed to soak for 24 hours to prepare a stock solution for bioassay. Bioassays were conducted by laying out 5 µL of the stock solution or a 10x dilution of the solution in a narrow line 2 cm long on a paper card and observing the response of a first-instar caterpillar to the extract. The study was replicated 10 times for each dilution using different caterpillars and different strips.

### Chemical analysis of mandibular gland extract, and response of caterpillars to fractionated components and to 2-[(9Z)- octadec-9-enoyl]cyclohexane-1,3-dione

**Figure 6.**
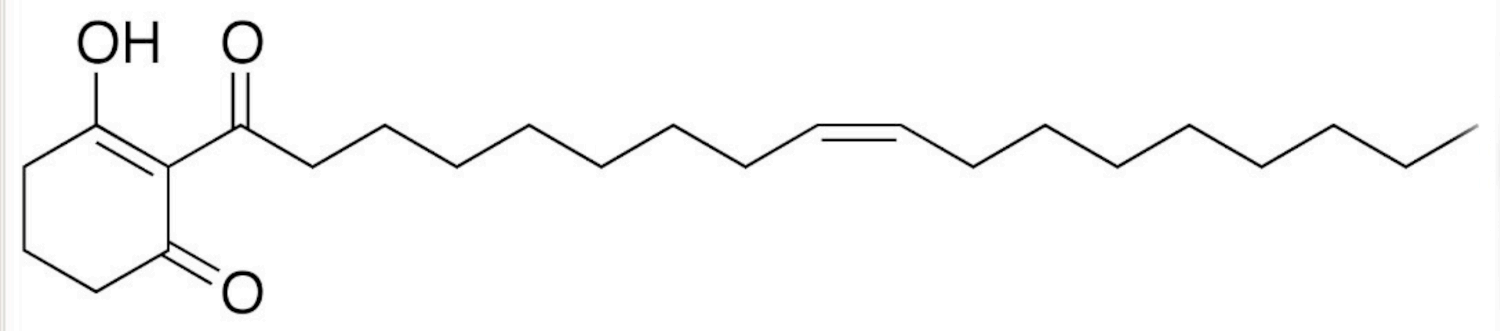
2-[(9Z)-octadec-9-enoyl]cyclohexane-1,3-dione. High quality figures are available online.

HPLC of the mandibular gland fluid was performed on a Dionex P580 instrument ( www.dionex.com ) equipped with a PDA-100 diode array detector and a Phenomenex Luna C8(2) column (100 x 4.6 mm, 5 µm pore size; ( www.phenomenex.co ). The hexane extract of 15 mandibular gland pairs was prepared as described above. The hexane was evaporated, and the resulting oil was diluted with methanol to a concentration of 10 caterpillar equivalents per mL. A 20 µL aliquot was injected into the HPLC and eluted with water (solvent A) and acetonitrile (solvent B) using the following solvent gradient: 30% A, 70% B to 10 % A, 90% B, for 10 min; 10% A, 90% B, for 10 min; 10% A, 90% B to 30% A 70% B, for 5 min; flow rate 1 mL/min. The fluid was split into seven fractions by HPLC (2.5–9 min, 9–10.5 min, 10.5–12 min, 12–14.4 min, 14.4–16 min, 16–17.5 min, 17.5–25 min). Each fraction was concentrated to 10 mandibular gland-pair equivalents per mL of solvent then bioassayed by laying out 5 µL of each extract as a narrow line (2 cm long) on a paper card and observing the response of first-instar caterpillars to the artificial trails. The response of caterpillars to 2-[(9Z)-octadec-9- enoyl]cyclohexane-1,3-dione ( Figure 6 ) was also tested. The chemical was synthesized using the procedure of Lakhvich et al. (2008) and assayed for purity by HPLC. To bioassay the chemical, 1 mg was dissolved in 1 mL of hexane. The response of first instar caterpillars to 5 µL quantities of the solution, laid out in lines 2 cm long on paper cards, was observed.

### Statistical Analysis

Chisquared analyses as detailed below were carried out with ProStat software (( www.polysoftware.com/prostat.htm ).

## Results

### Trail following behavior

In Y-maze tests, all of the 10 caterpillars chose the arms of mazes constructed from paper strips previously walked on by conspecifics over blank strips, showing that the caterpillars deposited a trail as they moved over the substrate that elicited following from conspecifics. The caterpillars were observed to swing their heads between the alternative pathways at the choice points before moving onto the selected strips. Such behavior, indicative of both klinotaxis and tropotaxis, has been commonly observed in other trail following caterpillars ( Peterson and Fitzgerald 1991 ). The behavior has been shown to allow caterpillars to compare alternative pathways at juncture points with their maxillary chemoreceptors.

### Persistence of the trail marker

In all Y-maze tests in which caterpillars were allowed to choose between blank paper strips and strips marked by conspecifics 0, 24, 48, and 120 hours previously, the caterpillars choose the marked strips. Although the caterpillars moved more slowly with frequent turn- backs on the most aged strips, all 14 of the tested caterpillars selected the previously marked strips for all age classes.

### Response of caterpillars to silk

When allowed to choose between paper strips bearing silk wound from the spinnerets of conspecifics and blank strips, six of 10 caterpillars chose the silk-marked strips (chisquared test, *Χ* 2 = 0.40, *P* = 0.53). Caterpillars moved hesitantly over the mazes and turned back frequently. When allowed to choose between paper strips previously moved over by caterpillars with cauterized spinnerets and blank strips, all of 12 caterpillars chose the former strips. Collectively, these two experiments indicate that silk in itself does not elicit trail following and is not a requisite component of pathways that elicit trail following behavior from the caterpillars.

### Response of caterpillars to cuticular residue

None of the 10 caterpillars tested followed artificial trails that had been prepared by stroking the creased edge of folded filter paper along the ventral surfaces of other caterpillars, nor did they show any response to the three unmarked creases. This indicates that unlike most other species of trail marking caterpillars, the caterpillars of *C. cactorum* do not mark trails with cuticular residue deposited by brushing the undersides of their bodies along the substrate.

### Response of caterpillars to whole body extract

All of the 10 caterpillars readily followed artificial trails prepared with the whole-body extract ( Video 1 ) but showed no response to solvent control trails. When allowed to choose in Y-maze tests between the arms of mazes bearing silk wound directly from a caterpillar’s spinneret and those marked with the extract, 18 of 20 caterpillars chose the arms marked with the extract ( *X* 2 = 12.8, *P* < 0.001), providing additional evidence that silk does not elicit a trail following response.

**Video 1.** Response of a first-instar *Cactoblastis cactorum* caterpillar to a whole body extract applied to a narrow strip of a paper card. Available online at: ( www.insectscience.org/14.64/video1.html

### Response of caterpillars to crude mandibular gland extract

**Figure 2.**
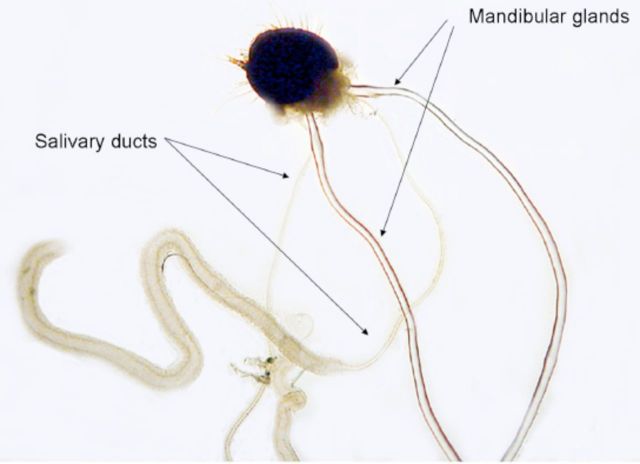
Mandibular glands of *Cactoblastis cactorum* . High quality figures are available online.

**Figure 3.**
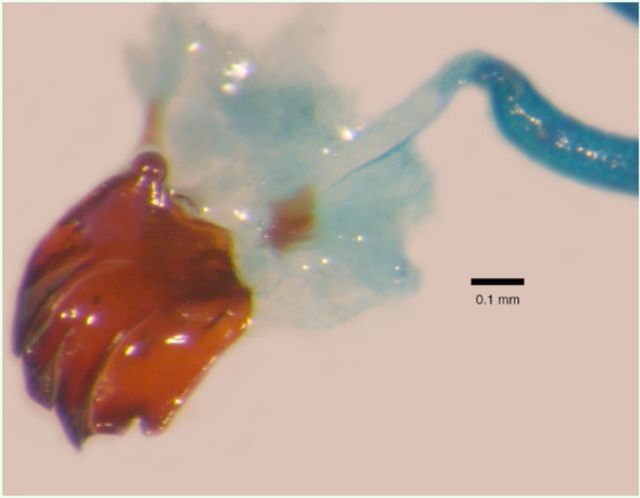
Stained preparation of the duct of the mandibular glands of *Cactoblastis cactorum* inserted into the apodeme of the adductor muscle of the mandible. High quality figures are available online.

Dissection of caterpillars showed that the larvae of *C. cactorum* have prominent paired mandibular glands filled with an oily liquid ( Figure 2 ). In fifth-instar caterpillars, the glands have a diameter of approximately 0.1 mm where they exit the back of the head. The glands extend far back into the abdomen, where they taper to very fine endings. When dissected and fully extended, the glands are approximately 2.5x the length of the caterpillar. The anterior end of each gland narrows to a duct approximately 0.4 mm long, which terminates in the apodeme of the adductor muscle of the mandible ( Figure 3 ). Studies of the mandibular glands of other pyralids indicate that the contents of the mandibular gland are secreted into the lumen of the apodeme and exit to the outside through an opening in the apodeme that occurs mesally at the base of the mandible ( Vegliante and Hasenfuss 2012 ).

**Figure 4.**
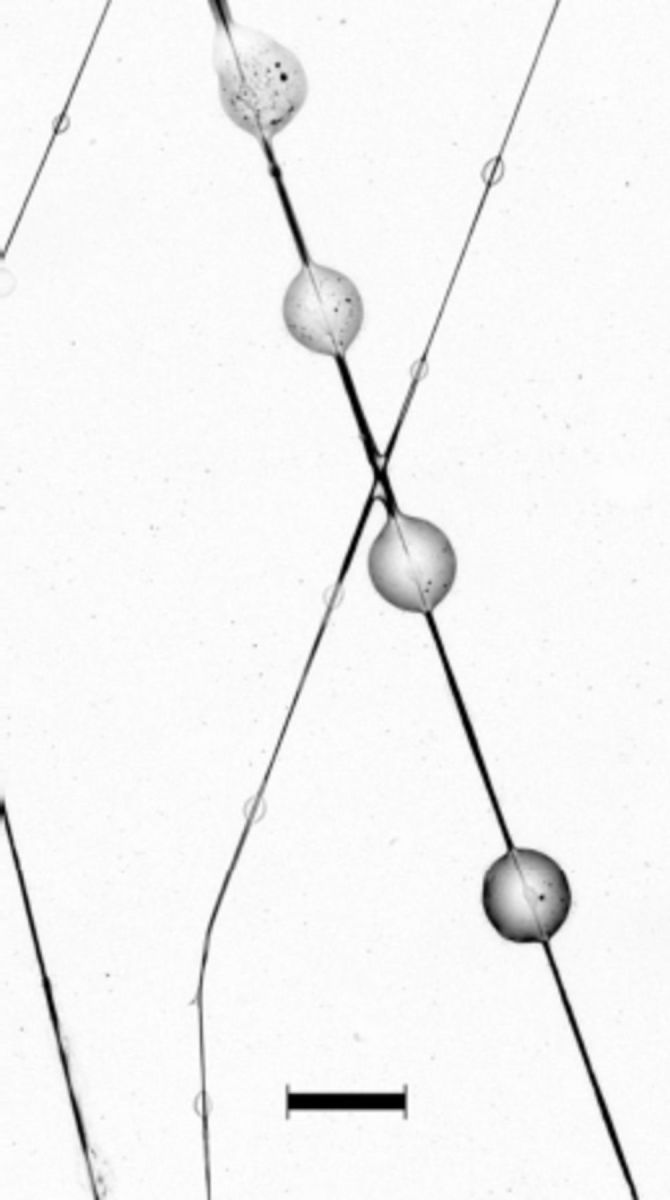
Large droplets of the mandibular gland secretion of *Cactoblastis cactorum* deposited on a silk strand. The small oval areas that appear on the silk stands in the background are not droplets but areas of nonlinearized silk, which appear to assist in tacking stands to the substrate. Scale = 250 µm. High quality figures are available online.

The mean wet mass of caterpillars from which glands were extracted in the present study was 99 ± 5 (SE) mg. The mean mass of a pair of glands from which water was allowed to evaporate for one hour was 1 ± 0.2 mg, or 1.19 ± 0.11% of the wet body mass of the caterpillars. The contents of the glands are transferred to the substrate when the undersurface of a caterpillar’s head brushes the substrate. The caterpillars were also observed to intermittently deposit oily droplets in a bead-like fashion along short lengths of the silk as the strands exited the spinneret ( Figure 4 ), a phenomenon similar to that previously described from the pyralid caterpillar *Plodia interpunctella* ( Mossadegh 1978 ). That silk pulled directly from the spinneret, as described above, did not elicit trail following, indicated that the caterpillars actively control the secretion of the mandibular gland oils.

All of the 10 caterpillars readily followed artificial trails prepared from the stock extract of the mandibular glands but showed no response to solvent control trails. The caterpillars, however, moved more slowly on strips marked with the 10x dilution of the extract and turned back frequently. When the amount of the diluted extract applied was increased from 5 µL to 10 µL, the caterpillars were more responsive, and all 10 followed the trails for their whole length but were still more hesitant than caterpillars tested on artificial trails marked with the stock solution of the extract. Artificial trails prepared with 5 µL of the stock solution incorporated the equivalent of 0.25% of the mean extractable contents of the paired glands of a caterpillar per mm of trail. However, because of the absorbency of the paper strips to which the extract was applied, only a fraction of this amount would reside on the surface where it could be detected by a caterpillar’s contact chemoreceptors. Thus, the behavioral threshold of the pheromone when applied directly to the substrate by the caterpillars is likely to be significantly lower than this. Later instars of *C. cactoblastis* were more agitated when handled than the first-instars and were not amenable to a trail bioassay of the type reported above. However, when the stock mandibular extract was laid out in long lines on a sheet of paper at the rate of 2.5 µL/cm to form artificial trails, caterpillars allowed to encounter the trails *ad libitum* often followed them for their whole length ( Video 2 ).

**Video 2.** Response of a fifth-instar *Cactoblastis cactorum* caterpillar to an artificial trail prepared from mandibular gland extract. The pencil dots show the location of the trail. Available online at: ( www.insectscience.org/14.64/video2.html

Observations of cohorts of first- to third-instar caterpillars placed on sheets of paper showed that the pathway of a single individual striking off on its own typically did not elicit a following response from other caterpillars. The deposition of a persistent pheromone trail appears to require the collective effort of small bands of caterpillars that advance over the substrate in close proximity or the repeated back and forth movement of a single individual over the same pathway. Although Dodd (1940) stated that the caterpillars he observed moved over the outside of the plant “…roughly in a single file,” *C. cactorum* is not a processionary caterpillar. Caterpillars reference the trail pheromone when traveling along a common pathway and do not maintain nor require direct physical contact with precedent individuals.

### Chemical analysis of mandibular gland extract and response of caterpillars to fractionated components

**Figure 5.**
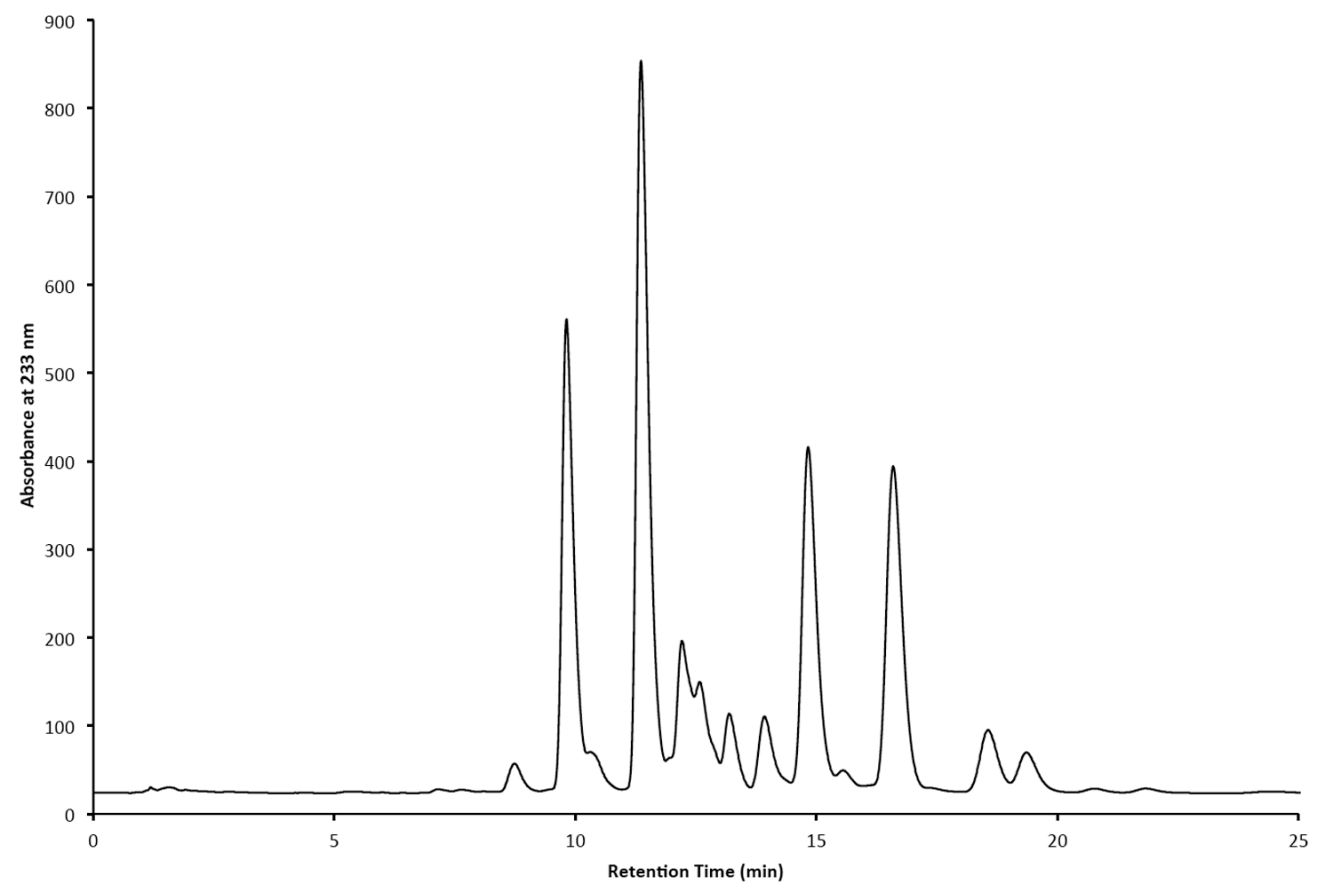
HPLC of the mandibular gland extract of *Cactoblastis cactorum* . High quality figures are available online.

HPLC analysis of the mandibular gland extract showed the presence of multiple components with absorption maximum at 233 ( Figure 5 ). Although the determination of the exact chemical structure of individual components of the mixture was beyond the scope of the present study, the dual UV-vis absorptions at 233 and 274 nm are consistent with the mixture containing a series of 2-acyl-1,3 cyclohexane diones similar to those identified by Mudd (1981) from the mandibular glands of *Ephestia kuehniella* Zeller. Caterpillars followed artificial trails prepared from all seven of the fractions of the whole extract but did not follow solvent control trails. There exists the possibility that the chemoreceptors of the caterpillars respond specifically to a molecular configuration shared by the components or, alternatively, that they are sensitive to a range of similar but distinct chemicals, as previously shown for the eastern tent caterpillar ( Crump et al. 1987 ). The oily nature of these compounds is attributable to the 16 or 18 carbon fatty acid side chains of the 2-acyl-1,3 cyclohexane diones. Indeed, artificial trails prepared with 2-[(9Z)-octadec-9- enoyl]cyclohexane-1,3-dione ( Figure 6 ) were readily followed by the caterpillars. Additional studies to identify, synthesize, and bioassay specific mandibular gland components are ongoing.

## Discussion

The study reported here shows that the caterpillars of *C. cactorum* mark their pathways with a secretion from their mandibular glands. Although silk is a conspicuous component of the caterpillars’ pathways, it does not elicit trail following behavior and is likely to function primarily to enhance steadfastness on the waxy surfaces of the host leaf. Dodd’s (1940) report, as cited in the introduction to this paper, is the only recorded observation of group movement of the caterpillar under field conditions. Thus, the functional significance of trail-based communication in this species is unstudied, but from what is known of the insect’s behavioral biology, trail marking is likely to be of adaptive value in several contexts. Pheromone trails may be essential to the maintenance of group cohesion during the intermittent surface forays that colonies of caterpillars have been reported to make following the depletion of a cladode ( Dodd 1940 ; Pettey 1947 ; Zimmerman et al. 2004). Colonies also leave cladodes temporarily to bask in the sun or cool in the shade ( Dodd 1940 ; Pettey 1947; Zimmerman et al. 2004), and trail marking may enable the caterpillars to relocate their entrance holes. At the time of hatching, the pheromone may also serve as an arena marker, constraining the movement of the neonate caterpillars to the vicinity of their egg mass. This would assure not only that the caterpillars stay together, but also that there are sufficient numbers in proximity to enable the successfully penetration of the plant, a task reported to involve a cooperative effort ( Dodd 1940 ).

While the mandibular gland fluids of other species of Pyralidae occurring in the same subfamily to which *C. cactorum* is assigned (Phycitinae) have been shown to act as semiochemicals, effecting both interspecific and intraspecific behavior, the present study is the first to report their use as a trail pheromone. Mossadegh (1980) found that the larvae of *P. interpunctella* preferred clean food to food marked with the mandibular gland secretion, and Phillips and Strand (1994) determined that substrates contaminated by the larvae elicited oviposition behavior from the moth for as long as 30 days after they were marked. Corbet (1971) studied the flour moth *E. kuehniella* and found that when larvae met, they deposited a mandibular gland secretion, which served as an epideictic pheromone that promoted larval spacing. Corbet (1973) also determined that the mandibular gland secretion of the larvae stimulated oviposition by the adult moth. Although none of the previously studied pyralid caterpillars have been reported to follow trails, the mandibular gland secretions of *E. kuehniella* have been shown to function as a kairomone, eliciting a following response from the parasitoid *Bracon hebetor* ( Strand et al. 1989 ). This wasp is also reported to attack the larvae of *C. cactorum* ( Dodd 1940 ), and the caterpillar’s mandibular gland secretions may be similarly exploited by the parasitoid.

The morphology and chemistry of the mandibular glands of several species of pyralids have been investigated. The glands of *E. kuehniella, E. cautella, E. elutella* , and *P. interpunctella* contain a clear, oily fluid that is secreted in copious quantities as the caterpillars move over the substrate (Mudd and Corbet 1973 ). Mudd (1978 , 1981, 1983) identified seven isomers of 2-acyl-1,3 cyclohexanedione and nine of 2-acyl-4- hydroxy-1,3 cyclohexanedione from the glands of *E. kuehniella* . Mossadeigh (1980) and Howard and Blake (2004) identified similar compounds from the mandibular glands of *P. interpunctella* . Mossadegh (1978) observed that the larvae of *P. interpunctella* deposit the oils as droplets, in bead-like fashion, along silk as the strands exit the spinneret, and also directly on the substrate. Both this secretory behavior and the mandibular gland itself, as indicated by a figure and measurements provided by Howard and Blake (2004) , closely resemble that of *C. cactorum.* 2-acyl-1,3 cyclohexanediones similar to those found in pyralid caterpillars have also been found in *Peperomia proctorii* (Piperaceae), but their function in the plant is unknown ( Seeram et al. 2000 ).

Based on Mossadeigh’s (1978) observations, Howard and Blake (2004) estimated, conser- vatively, that the larva of *P. interpunctella* secretes approximately 3.25 mg of the mandibular gland oils during its lifetime. As reported here, *C. cactoblastis* also secreted large quantities of oils, though the exact amounts remain to be determined. Since trail pheromones of insects are typically secreted in microgram or nanogram quantities, the use of mandibular gland oils by *C. cactorum* to mark pathways suggests that their use in this context may be derivative. Howard and Blake (2004) found that in the larvae and pupae of *P. interpunctella* , the oily secretions serve to waterproof the cuticle. The cuticular lipids of this species are dominated by approximately twenty different 2-acyl-1,3-cyclohexanediones derived from the mandibular glands. The caterpillars became coated with the secretion as they moved over substrates contaminated with the oily material. If this were the primary function of the secretion, then it might explain why Phycitinae caterpillars, including *C. cactorum* , produce so much of it. However, this phenomenon has not yet been reported in species other than *P. interpunctella* . In the present study of *C. cactorum* , caterpillars failed to respond to surface residues from the ventral surface of the body, indicating that the mandibular gland secretion does not collect there in quantities sufficient to elicit trail following. Another explanation for the copious production and marked isomerism of the 2- acyl-1,3-cyclohexanediones is suggested by the observation that similar compounds have insecticidal, fungicidal, and antimicrobial properties ( Mudd 1981 ). However, there is as yet no evidence in support of this possibility.

With the inclusion of the Pyralidae, social caterpillars in seven families of the Lepidoptera have now been shown to mark and follow trails ( Pescador-Rubio et al. 2011 ). Most of the previously studied species of lepidopterous caterpillars ( Fitzgerald 1995 ) and the larvae of the weevil *Phylepera distigma* (Coleoptera; Costa et al. 2004 ; Fitzgerald et al. 2004 ) mark trails by brushing their pheromones onto the substrate from the ventral surfaces of their abdomens. In *Hylesia linea- tea* (Saturniidae), the source of the pheromone has been tentatively identified as glandular hairs that arise from the ventral surface of the last abdominal segment ( Fitzgerald and Pes- cador-Rubio 2002 ). In the lasiocampid caterpillars *Malacosoma* spp. ( Fitzgerald 1995 ) and *Eriogaster lanestris* ( Ruf et al. 2001 ), the pierid *Eucheria socialis* ( Fitzgerald and Underwood 1998 ), and the saturniid *Arsenura armada* ( Costa et al. 2001 ), the pheromones are transferred to the substrate when the caterpillars lower and drag the ventral surfaces of their last abdominal segments along the substrate. No definitive glands have been identified from this region of the abdomen, and it has been hypothesized for the lasiocampids that the pheromone is a product of epidermal secretory cells ( Fitzgerald 1995 ; Ruf et al. 2001 ). Trail marking associated with silk has been reported from *Archips cerasivoranus* ( Fitzgerald 1993 ), *Yponomeuta cagnella* ( Roessingh 1989 ), and *Hemileuca oliviae* ( Capinera 1980 ), but the possible involvement of a gland other than the silk gland has not been investigated in any of these species. To our knowledge, the present study is the first report of any insect marking terrestrial trails with an extra-silk secretion that empties at the anterior end of the body. Stingless bees, however, may create aerial trails by marking substrate intermittently with labial gland secretions to guide the flight of foragers ( Nieh et al. 2004 and references therein). Ants and termites, the preeminent trail makers, secrete trail pheromones from openings that occur on the abdomen or hind legs ( Wilson 1971 ; Hölldobler and Wilson 1990 ; Billen 2009 ).
